# Dynamic Force Production Capacities Between Coronary Artery Disease Patients vs. Healthy Participants on a Cycle Ergometer

**DOI:** 10.3389/fphys.2019.01639

**Published:** 2020-01-24

**Authors:** Marie Fanget, Jérémy Rossi, Pierre Samozino, Jean-Benoît Morin, Rodolphe Testa, Frédéric Roche, Thierry Busso, Jari Antero Laukkanen, David Hupin

**Affiliations:** ^1^UJM-Saint-Etienne Autonomic Nervous System Research Laboratory, EA 4607 SNA-EPIS, University of Lyon, Saint-Étienne, France; ^2^UJM-Saint-Etienne Interuniversity Laboratory of Human Movement Biology, EA 7424, University of Lyon, Saint-Étienne, France; ^3^Laboratoire Interuniversitaire de Biologie de la Motricité, EA 7424, University Savoie Mont Blanc, Chambéry, France; ^4^LAMHESS, University of Côte d’Azur, Nice, France; ^5^Department of Clinical and Exercise Physiology, University Hospital of Saint-Etienne, Saint-Étienne, France; ^6^Institute of Public Health and Clinical Nutrition, University of Eastern Finland, Joensuu, Finland; ^7^Department of Internal Medicine, Central Finland Central Hospital, Jyväskylä, Finland; ^8^Faculty of Sport and Health Sciences, University of Jyväskylä, Jyväskylä, Finland

**Keywords:** force-velocity-power relationship, cardiac rehabilitation, physical activity, acute coronary syndrome, cycle sprint, exercise physiology, health

## Abstract

**Background:**

The force-velocity-power (FVP) profile is used to describe dynamic force production capacities, which is of great interest in training high performance athletes. However, FVP may serve a new additional tool for cardiac rehabilitation (CR) of coronary artery disease (CAD) patients. The aim of this study was to compare the FVP profile between two populations: CAD patients vs. healthy participants (HP).

**Methods:**

Twenty-four CAD patients (55.8 ± 7.1 y) and 24 HP (52.4 ± 14.8 y) performed two sprints of 8 s on a Monark cycle ergometer with a resistance corresponding to 0.4 N/kg × body mass for men and 0.3 N/kg × body mass for women. The theoretical maximal force (*F*_0_) and velocity (*V*_0_), the slope of the force-velocity relationship (*S*_fv_) and the maximal mechanical power output (*P*_max_) were determined.

**Results:**

The *P*_max_ (CAD: 6.86 ± 2.26 W.kg^–1^ vs. HP: 9.78 ± 4.08 W.kg^–1^, *p* = 0.003), *V*_0_ (CAD: 5.10 ± 0.82 m.s^–1^ vs. HP: 5.79 ± 0.97 m.s^–1^, *p* = 0.010), and *F*_0_ (CAD: 1.35 ± 0.38 N.kg^–1^ vs. HP: 1.65 ± 0.51 N.kg^–1^, *p* = 0.039) were significantly higher in HP than in CAD. No significant difference appeared in S_fv_ (CAD: −0.27 ± 0.07 N.kg^–1^.m.s^–1^ vs. HS: −0.28 ± 0.07 N.kg^–1^.m.s^–1^, *p* = 0.541).

**Conclusion:**

The lower maximal power in CAD patients was related to both a lower *V*_0_ and *F*_0_. Physical inactivity, sedentary time and high cardiovascular disease (CVD) risk may explain this difference of force production at both high and low velocities between the two groups.

## Introduction

After an acute coronary syndrome, a cardiac rehabilitation (CR) program is essential to restore or increase physical capacities and reduces cardiovascular disease (CVD) risk (Pavy et al., 2012; Iliou et al., 2015; Price et al., 2016). The objective for active subjects is to regain their place in society and for older persons is to maintain their independence (Pavy et al., 2012; Iliou et al., 2015). It is necessary to adapt the content of CR sessions to optimize aerobic and anaerobic performance along with quality of life (Price et al., 2016).

Specifically, the improvement of maximum power output of the neuromuscular system is one of the objectives sought in CR. Muscle power (*P*), which is the product of force (*F*), and velocity (*V*), is essential to enhance anaerobic performance (Cronin and Sleivert, 2005; Morin and Samozino, 2016). Maximal power capacities depend on force production abilities over the entire spectrum of contraction velocities, which can be well described by the force-velocity (FV) relationship (Morin and Samozino, 2016). The orientation of this FV relationship toward rather maximal force at low velocities (i.e., force capacity) or force at high velocities (i.e., velocity capacity) is well characterized by its slope, which refers to the FV profile (Giroux et al., 2016).

Several studies have been carried out on the force-velocity-power (FVP) relationship and sport performance in top athletes (Samozino et al., 2014a, b; Giroux et al., 2016; Morin and Samozino, 2016). This FVP profile can be evaluated on ballistic push-offs and sprint movements (on treadmill or cycle ergometer) (Seck et al., 1995; Morin et al., 2010). Different profiles may be determined according to the type of physical activity and sometimes even the athlete’s position (e.g., toward force capacity for forward players and toward velocity for back players in rugby) (Morin and Samozino, 2016). The analysis of the FVP profile highlights the weaknesses in force production capacity of each athlete. A specific training oriented in force or in velocity should be adapted according to whether the athlete wants to maintain his/her specificity or tip the balance of the FV profile that presents a deleterious imbalance for his/her future performances (i.e., change the slope of the right of the FV profile toward an optimal slope) (Jiménez-Reyes et al., 2016; Morin and Samozino, 2016).

Optimizing the exercise training program is constantly sought in rehabilitation among patients always younger with coronary artery disease (CAD) (Price et al., 2016). Indeed, the CAD prevalence rose from an estimated 290 cases per 100,000 for those 40–44 years of age to 11,203 cases per 100,000 at 1990–2015 (Roth et al., 2017). Therefore, it might be interesting to talk about performance even in patients and to use the FVP relationship to more precisely identify this loss of muscle force production capacity in patients suffering from cardiovascular impairment as well a loss of functional capacities of their neuromuscular system. Usual rehabilitation sessions are based on the results of functional explorations performed in aerobic (cardiorespiratory exercise test) and resistance (static and dynamic quadriceps test) at the beginning of the rehabilitation cycle. Instead of using the results of a muscle test, we could rely on the results of the initial FVP. So far, the measurement of muscle strength production capacities has been determined by isometric and dynamic leg extension test. The FVP profile would allow, through a simple and rapid assessment, to target the weakest qualities in patients in order to individualize the training for each person.

The aim of the present study was to analyze and compare the force production capacities through the mechanical variables of the FVP relationship obtained during pedaling between CAD patients and healthy participants (HP). We hypothesized that CAD patients present lower maximal power than HP, with notably a lower maximal force production at low velocity; but without previous studies, we had no evidence to suggest the impact of FV profile (i.e., whether one would be more affected than the other).

## Materials and Methods

### Participants

Coronary artery disease patients volunteered to participate in this study at the beginning of the CR. The inclusion criteria were the following: (a) over 18 years of age; (b) received medical treatment and percutaneous coronary intervention (angioplasty with stent implantation) or surgical revascularisation (coronary artery bypass grafting); and (c) maximal aerobic power superior to 60 W for women and 80 W for men (Borjesson et al., 2011). They received a measurement of their maximal oxygen uptake ($\dot{\text{V}}$O_2_max) during an ergocycle stress test before and after a CR program. The study protocol conformed to the ethical guidelines of the 1975 Declaration of Helsinki and was approved by the institution’s human research committee. The study was registered in the National Institutes of Health ClinicalTrials.gov database.

HP were 18 years old as patients, they were free from any kind of CVD, performing regular physical exercise and were volunteers to participate. The ethics committee (*IRBN372016/CHUSTE*) and the national commission for informatics and liberties (*CNIL165853*) approved the cohort study for pedaling testing.

History of sport was assessed by only two questions: (1) did you do sports (CAD patients) or (2) do you practice sports (HP)? If yes, which sport? Sport was quantified in weekly metabolic equivalent of task (MET) and expressed in h/week (i.e., intensity in MET × duration × frequency), using the compendium of sports, and ranged from 6 (vigorous intensity = sport) to 18 MET (running at 17 km/h) (Ainsworth et al., 2000).

### Experimental Protocol

**FIGURE 1:**
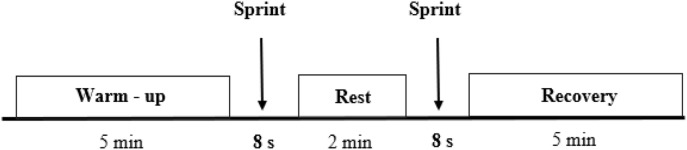
Protocol diagram.

At the beginning of each experiment, saddle height was adjusted and toe clips were well fastened to avoid losing the pedals. After a 5-min warm-up, participants performed two maximal 8-s duration sprints, separated by a 2-min rest period, against friction loads of 0.4 and 0.3 N.kg^–1^ body mass for men and women, respectively (Figure 1). We performed pre-tests to determine these appropriate loads. They had to remain seated during the test. For each trial, the participants had to pedal as fast as possible during all the sprint. For this, the experimenter (MF) encouraged vigorously each participant throughout the sprint. We retained the data of the best sprint (i.e., the one with the highest maximal power).

### Material

**FIGURE 2:**
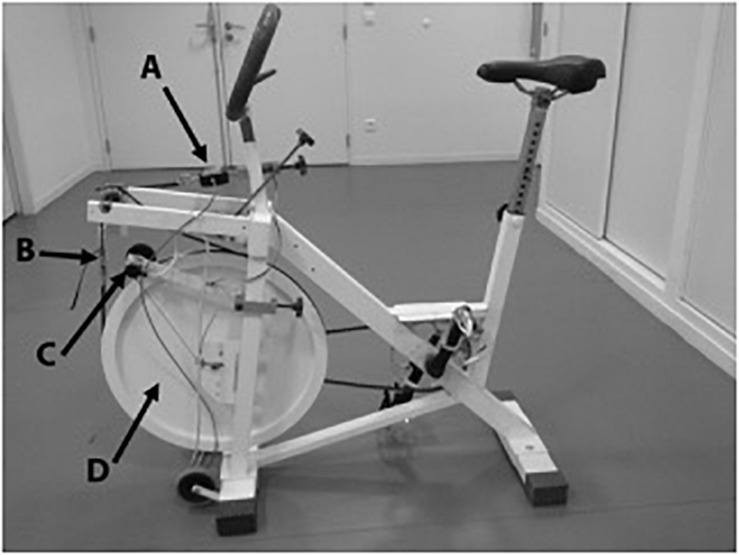
Friction loaded Monark cycle ergometer. A, Strain gauge; B, Belt; C, Optical encoder; D, flywheel.

A friction-loaded cycle ergometer (Monark, Vansbro, Sweden) was used (Figure 2). All features of the ergometer were detailed in previous studies (Arsac et al., 1996; Morin and Belli, 2004). The apparatus was instrumented with a strain gauge (FGP Instrumentation, FN 3030 type, Les Cloyes Sous Bois, France) to measure the friction force applied by the tension of the belt and an optical encoder (Hengstler type RI 32.0, 100 pts/turn, Aldingen, Germany) to measure the flywheel displacement. The inertia was determined from the linear relationship obtained by free deceleration of the flywheel. Data were sampled at 200 Hz and recorded in LabVIEW software. Data were filtered with a 4th order low pass Butterworth filter at 30 Hz. Angular velocity and pedaling frequency were calculated from filtered displacement.

### *F*-*V* Relationship

The power output (*P* in watts) produced at each instant during the sprint was computed as follows (Morin and Belli, 2004):

(1) P=(F+frictF)inert×V,

where *F*_frict_ was the friction force, *F*_inert_ the inertial force (computed from the flywheel inertia and acceleration) and *V* the flywheel linear velocity. Instantaneous flywheel linear velocity was calculated from the flywheel displacement. The force (*F* = *F*_frict_ + *F*_inert_), power (*P*) and velocity (*V*) variables corresponded to mechanical outputs at the flywheel (Jiménez-Reyes et al., 2016).

**FIGURE 3:**
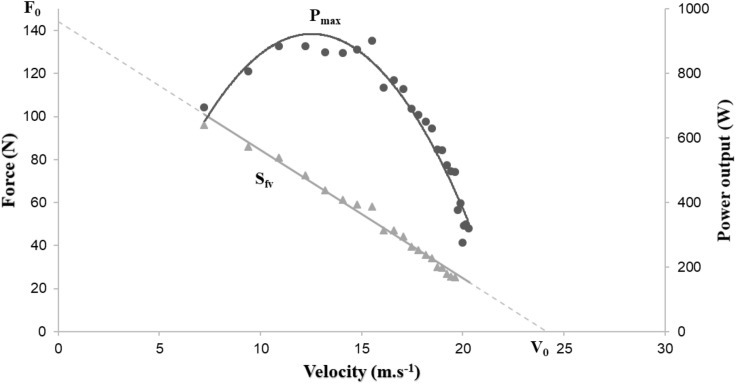
Graphical representation of power-force-velocity relationship.

The *F*, *V*, and *P* values were averaged for each pedal downstroke, which were defined between two successive minimal values of instantaneous power (Samozino et al., 2007). The lower limb force production capacities can be described by the negative linear relationship (*F*-*V*) and the second order polynomial relationship (*P*-*V*). From these two relationships, a few parameters, which reflected the mechanical limits of the neuromuscular system, can be determined (Figure 3; Driss et al., 2002; Morin and Samozino, 2016): the theoretical maximum force (*F*_0_) which could be developed at zero velocity (intercept value on the *y*-axis); the theoretical maximum velocity (*V*_0_) until which force could be produced (intercept value on the *x*-axis); and the maximum power output (*P*_max_), corresponding to the maximum power that an individual is able to develop and the slope of *F*-*V* relationship (*S*_fv_) which can be computed as follows:

(2) P=max(F×0V)0/4,

(3) S=fv-F/0V,0

### Statistical Analysis

All data were expressed as mean ± standard deviation (SD). After checking distribution of normality with the Shapiro–Wilk test, Student’s t-tests for independent groups were used to detect differences in *F*_0_, *V*_0_, *S*_fv_, and *P*_max_ between the two populations. For all analyses, statistical significance was defined as *p* < 0.05. Cohen’s d was also computed to indicate the effect size, which corresponded to the difference between two means divided by the pooled SD. We interpreted the data obtained in this way: <0.2 was trivial, 0.2–0.5 a weak effect, 0.5–0.8 a medium effect, and >0.8 a strong effect (Parker and Hagan-Burke, 2007).

## Results

Twenty-four CAD patients and 24 HP aged 55.8 (± 7.1) y and 52.4 (± 14.8) y, respectively, participated in this investigation. Overweight (mean BMI > 25 kg/m^2^) concerned 54% of the CAD patients and they had significantly higher BMI than HP (27.5 ± 5.4 vs. 24.4 ± 3.4 kg/m^2^, *p* < *0.05*). In addition, these patients were physically inactive (5.3 ± 6.8 vs. 39.7 ± 42.0 MET-h/week, *p* < 0.001). Descriptive characteristics of participants are presented in Table 1.

**TABLE 1:** Morphological characteristics of participants.

**Variable**	**CAD Patients *n* = 24 (*7 Females/17 Males)***	**Healthy Participants *n* = 24 *(8 Females/16 Males)***	***p*-value**
Age (y)	55.8 ± 7.1	52.4 ± 14.8	0.319
**Body mass (kg)**	81.5 ± 17.1*	**71.9 ± 13.6**	**0.036**
Height (cm)	172 ± 9	171 ± 10	0.864
**BMI (kg/m**^2^)	27.5 ± 5.4*	**24.4 ± 3.4**	**0.020**
**PA (MET-h/week)**	5.3 ± 6.8***	**39.7 ± 42.0**	**0.000**

BMI, body mass index; PA, physical activity; CAD, coronary artery disease; MET, metabolic equivalent of task. Values presented as means ± SD. Statistical difference between two groups *(*p* < 0.05), ***(*p* < 0.001).

**FIGURE 4:**
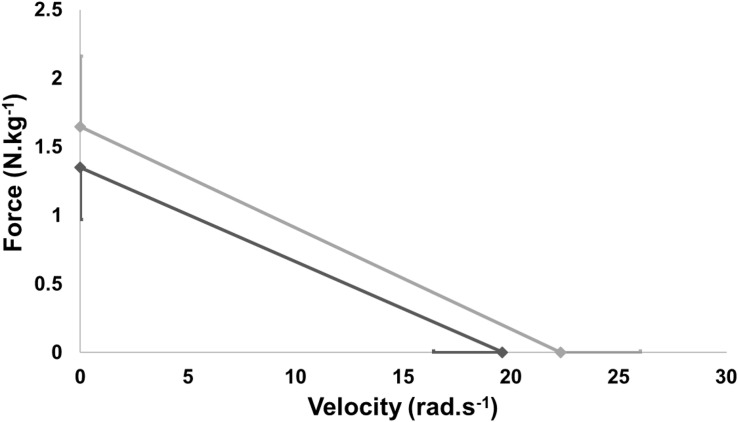
Mean force-velocity profile of healthy subjects and coronary patients.

The average of mechanical parameters of the FVP for each group are reported in Table 2. We noted a significant difference for *P*_max_, *V*_0_, and *F*_0_. A smaller in *P*_max_ (−29.8%, *p* = 0.003), *V*_0_ (−11.9%, *p* = 0.010), and *F*_0_ (−18.2%, *p* = 0.039) were observed in CAD patients compared to HP. However, no statistical difference was observed between the two populations for *S*_fv_ (*p* = 0.557) of FVP (Figure 4).

**TABLE 2:** Mechanical performance sprint variables.

**Variable**	**CAD Patients (*n* = 24)**	**Healthy Participants (*n* = 24)**	***p*-value**	**Cohen’s *d***
*F*_0_ (N)	106.84 ± 27.78	117.14 ± 43.43	0.333	0.283
***F*_0_ (N.kg**^–1^)	1.35 ± 0.38*	**1.63 ± 0.51**	**0.039**	**0.642**
***V*_0_ (m.s**^–1^)	5.10 ± 0.82*	**5.79 ± 0.97**	**0.010**	**0.722**
***V*_0_ (rad.s**^–1^)	19.6 ± 3.16*	**22.3 ± 3.73**	**0.010**	**0.726**
*S*_fv_ (N.kg^–1^.m.s^–1^)	−0.27 ± 0.07	−0.28 ± 0.07	0.541	0.142
***P*_max_ (W)**	543.47 ± 170.36*	**709.88 ± 328.14**	**0.032**	**0.612**
***P*_max_ (W.kg**^–1^)	6.86 ± 2.26**	**9.78 ± 4.08**	**0.003**	**0.816**

*F*_0_, theoretical maximum force; *V*_0_, theoretical maximum velocity; *S*_*fv*_, Slope of linear force-velocity relationship; *P*_*max*_, maximal power output; CAD, coronary artery disease. Values presented as means ± SD. Statistical difference between two groups *(*p* < 0.05), **(*p* < 0.01).

## Discussion

This study was the first to examine the mechanical parameters of FVP in CAD patients. The main findings of this research were that (i) *P*_max_, *V*_0_, and *F*_0_ were significantly lower in CAD patients than in HP, while (ii) *S*_fv_ was similar for these two populations.

### Decrease in *P*_max_, *F*_0_ and *V*_0_ on CAD Patients

Considering power output as the product of force and velocity (eq.1), the decline of maximum power was induced by a reduction of both force production capacities at high (*V*_0_) and low (*F*_0_) velocities. The values presented in the literature are mostly based on high performance athletes, which leads to higher results than those reported in our study (Vandewalle et al., 1987; Dorel et al., 2005). Dorel et al. (2005) and Vandewalle et al. (1987) measured peak power (*P*_max_) of elite cyclists who performed short maximal sprints (about 5–6 s) on a Monark cycle ergometer. The findings of these two studies were, respectively 19.3 ± 1.3 and 16.8 ± 1.23 W.kg^–1^, i.e., it was almost two and three times the maximum power developed, respectively, by HP and CAD patients.

This difference in force and velocity between the two groups can be explained by different factors. First, CAD patients were significantly fatter than HP and therefore had a higher BMI. In addition, we have no significant difference between the BMI in CAD patients before and after CR (Before CR: 27.6 ± 5.5 kg/m^2^ and after CR: 26.6 ± 5.0 kg/m^2^, *p* = 0.44). Secondly, prior to their acute coronary syndrome, patients were physically inactive. They spent on average 5 MET-h/week, which was very low knowing that 1 MET represented the metabolism at rest (3.5 ml O2/kg/min) (Jetté et al., 1990). Besides, it was recommended to walk on average 10,000 steps/d and to practice at least 150 min of physical activity weekly (Le Masurier et al., 2003) which represents an average dose of 7.5 MET-h/week (Hupin et al., 2015). We noted a significant difference on $\dot{\text{V}}$O_2_max in CAD patients before and at the end of CR (before CR: 22.70 ± 5.20 ml/min/kg and after CR: 25.82 ± 5.78 ml/min/kg, *p* = 0.0018). Thanks to CR, CAD patients improved their $\dot{\text{V}}$O_2_max by 15%. Compared to the reference values for people of the same age [i.e., 38.4 ml/min/kg (Wilmore et al., 2008)] this confirms that CAD patients are deconditioned before the CR program. Thirdly, patients often had more sedentary behavior before cardiovascular event. These risk behavioral factors, combined with CVD risk factors such as smoking and poor diet, significantly increase cardiovascular morbidity and mortality (Gbd 2013 Risk Factors Collaborators, 2015). We can suppose that high CVD risk and an excess fat mass prevented them from contracting at high velocities.

This difference of *V*_0_ might be due to a remodeling of the motor units toward a slower typology (Power et al., 2014). Other physiological factors could be involved, such as an increase internal resistance produced by connective tissue (Valour et al., 2003) an increase in percentage of type I fibres (Thom et al., 2007) and selective atrophy of type II fibers (Häkkinen et al., 1996). This last assumption was in accordance with (Hautier et al., 1998), who showed an important relationship between maximal power and the relative area of fast twitch fibers. Indeed, most HP practiced explosive sports such as football and tennis, which requested fast twitch fibers and anaerobic metabolism.

The variation in *F*_0_ might be caused by sarcopenia, the loss of muscle mass in patients (Häkkinen et al., 1996). In addition, most of the patients had sedentary behavior (i.e., overweight and physically inactive), which led to higher a percentage of fat tissue compared to HP. Thirdly, they might also have a neuromuscular activation deficit or the decrease in the effectiveness of the transmission of the voluntary command to the muscle could explain the decrease in force (Morse et al., 2004).

In addition, all CAD patients (except one) were receiving beta-blocker medical treatment (none among HP). These medications have the effect of decreasing heart rate and blood pressure (Goldberger et al., 2015). Beta-blockers would change neuromuscular recruitment strategy, which would explain the impaired maximal sprint performance (Hunter et al., 2002; Fisher et al., 2010). Moreover, statin therapy demonstrated a benefit in CAD patients to reduce CVD risk (Shepherd et al., 1995); however, they had deleterious effects on skeletal muscle, ranging from muscle complaints (which explained the withdrawal of statin in 2 CAD patients) to myositis (Mikus et al., 2013). Finally, the treatment of CAD patients (statin, beta-blockers), the disease and low physical activity had negative effects on muscle function.

### Similar Values of Slope

No significant difference was observed for the *S*_fv_ variable between the two groups. The *P*_max_ impacted both force production at high and low velocities. The current results differed with previous studies which indicate higher *F*_0_ values (Driss et al., 1998; Giroux et al., 2016). Indeed, Driss et al. (1998) assessed mechanical properties of FVP in male volleyball players during short maximal sprint (about 6 s) on a Monark cycle ergometer and they reported a *F*_0_ value almost two times higher than ours.

### Limitation

As physical activity was evaluated solely through a few questions, which is an approximate measure of the quantity of physical exercise, the main limitation of this study regards the objectivity of physical activity assessment. For future studies, more precise tools such as actimeters should be used.

### Perspectives

This study could be continued by a randomized study with a larger number of participants to assess the impact of training in force or velocity production force capacities according to the initial FVP of the patients. Indeed, evidence may be emerging that high-intensity strength training is more effective to increase acutely myofibrillar protein synthesis, cause neural adaptations and, in the long term, increase muscle strength, when compared to low-intensity strength training (Hansen et al., 2019). Also, studies report that cardiovascular demand is lower in high-intensity than low-intensity resistance exercises, thus potentially pointing toward sufficient medical safety of a simple sprint on cycle ergometer for the cardiovascular system (Bjarnason-Wehrens, 2019). The *F*-*V* (deficit in force or velocity) imbalance initially observed from an evaluation of the FVP (sprint on cycle ergometer) would be optimized thanks to an adapted training program. We hypothesize that *F*-*V* profile could be used in CR in CAD patients, as an additional and novel tool, to induce a *F*-*V* balance adapted through personalized sessions (Supplementary Material).

## Conclusion

The lower maximal power in CAD patients was related to both a lower *V*_0_ and *F*_0_. Physical inactivity, sedentary time and high CVD risk may explain this difference of force production at both high and low velocities between the two groups.

## Data Availability Statement

All datasets generated for this study are included in the article/Supplementary Material.

## Ethics Statement

The studies involving human participants were reviewed and approved by the French Ethics Committee. The patients/participants provided their written informed consent to participate in this study.

## Author Contributions

MF and DH contributed to the conception and design of the work. JR, PS, and TB contributed to the analysis and interpretation of the data. MF drafted the manuscript. JR, PS, J-BM, RT, FR, JL, and DH critically revised the manuscript. All authors gave final approval and agreed to be accountable for all aspects of work ensuring integrity and accuracy.

## Conflict of Interest

The authors declare that the research was conducted in the absence of any commercial or financial relationships that could be construed as a potential conflict of interest.
