# Supplementary material for: Dynamic Force Production Capacities Between Coronary Artery Disease Patients vs. Healthy Participants on a Cycle Ergometer
Source: Front Physiol. 2020 Jan 24;10:1639. doi: 10.3389/fphys.2019.01639 (PMC6993059; doi:10.3389/fphys.2019.01639)
Supplement: Supplementary file 1 [file Table_1.DOCX]

| **Bike** | VELOCITY GROUP | FORCE GROUP |
| --- | --- | --- |
| **Warm-up** | **10 min: HR=30-50% VT1** | |
| **Work** | **20 min** | |
| *Style* | Interval training  4x (1 min 30 HR=VT1/30s HR=VT2)  3 min HR=50-60%VT1  6x (1 min HR=VT1/30s HR=VT2) | Continued (HR=VT2) |
| *Velocity* | >100 RPM | <50 RPM |
| **Recovery** | **10 min: HR=30-50% VT1** | |
|  |  |  |
| **Walking** | VELOCITY GROUP | FORCE GROUP |
| **Warm-up** | **10 min: HR=30-50% VT1** | |
| **Work** | **20 min** | |
| *Speed* | High (VT1-VT2) | Low (<VT1) |
| *Slope* | 0 | High (5%) |
| **Recovery** | **10 min: HR=30-50% VT1** | |
|  |  |  |
| **Resistance** | VELOCITY GROUP | FORCE GROUP |
| **Warm-up** | **2 x 2** (20% MVC) | |
| **Work** | **8 x 8 --> 10 x 10** | **6 x 6 --> 8 x 8** |
| *Speed* | Quick execution | Slow execution |
| *Load* | Low (20-40% MVC) | High (60-80% MVC) |
| **Recovery** | **2 x 2** (20% MVC) | |

HR: Heart rate

VT1: ventilatory threshold 1

VT2: ventilator threshold 2

RPM: rate per minute

MVC: maximum voluntary contraction

A succession of 2 movements of muscular strengthening repeated 2 times at a low intensity (20% of maximum strength).

**Supplementary file.** Cardiac rehabilitation adapted program according to the weakness in force or in velocity of the CAD patients
